# Relationship between the higher inflammatory cytokines level in the aqueous humor of Fuchs uveitis syndrome and the presence of cataract

**DOI:** 10.1186/s12886-021-01860-3

**Published:** 2021-02-27

**Authors:** Hui Wang, Yong Tao

**Affiliations:** Department of Ophthalmology, Beijing Chaoyang Hospital, the Third Clinical Medical College of Capital Medical University, No. 8, Worker’s stadium south road, Chaoyang District, Beijing, 100020 PR China

**Keywords:** Fuchs uveitis syndrome, Aqueous humor, Cytokines, Cataract, Posterior subcapsular cataract

## Abstract

**Background:**

This study aims to compare the levels of intraocular cytokines between Fuchs uveitis syndrome (FUS) eyes and the senile cataract eyes. The association between inflammatory cytokine levels and cataract severity in FUS is evaluated to find the possible mechanism of cataract in FUS eyes.

**Methods:**

A retrospective study of 28 eyes with FUS was performed. Auxiliary examinations were performed, including ophthalmic examinations, laser flare-cell photometry, and levels of inflammatory cytokines in the aqueous humor were measured. The control group included 25 eyes with senile cataract. Data on the aqueous humor inflammatory cytokines were compared between the two groups. The association between the aqueous humor cytokine levels and severity of posterior subcapsular cataract was assessed.

**Results:**

There were 28 eyes with FUS in 27 patients. Unilateral involvement was noted in 26 patients (96.30%). Stellate keratic precipitates (KPs) were noted in 16 eyes (57.14%). Heterochromia was observed in 21.43% of affected eyes. Posterior subcapsular cataract (PSC) was observed in 16 of the 28 eyes. Eyes with FUS had significantly higher aqueous humor (AH) cytokine levels (VEGF, bFGF, IL-6, IL-8 and IL-10) compared with the control eyes (*P* < 0.05). There was a statistically significant positive correlation between the severity of cataract and IL-6 and IL-8 levels in the AH (τ = 0.664 and 0.634, respectively; *P* = 0.001, *P* = 0.002, respectively).

**Conclusions:**

Expression of VEGF, bFGF, IL-6, IL-8 and IL-10 in the AH of FUS patients was significantly higher than in senile cataract eyes, and the aqueous humor levels of IL-6 and IL-8 were significantly positively associated with the severity of posterior subcapsular cataract. Our results imply that an inflammation mechanism may be involved in the early development of cataract in FUS.

**Supplementary Information:**

The online version contains supplementary material available at 10.1186/s12886-021-01860-3.

## Background

Fuchs uveitis syndrome (FUS) is an intraocular inflammatory condition which was first described in 1906 by Fuchs [1]. FUS is an unilateral chronic recurrent non-granulomatous uveitis syndrome in about 90% of cases and accounts for 2 ~ 11% of uveitis, characterized by diffuse distribution of stellate keratic precipitates (KP), heterochromia, iris depigmentation, iris atrophy and early cataract formation [1]. Although the trigger mechanism of FUS is unknown, many studies have shown that immunological factors may play a role in the development of FUS [2–4]. However, few studies have compared inflammatory cytokine levels in the aqueous humor of FUS patients and normal eyes until now.

The most common complications in FUS patients are complicated cataract and glaucoma. Due to the long-term effects of inflammation, the average incidence of cataract in FUS is around 50% [5–8]. Cataract is a common complication of chronic or recurrent uveitis, which may be caused by intraocular inflammation, long-term use of corticosteroids, or a synergistic effect of both [9]. Vascular endothelial growth factor (VEGF) and pro-inflammatory cytokines play a role in the development of cataract in patients with non-infectious uveitis. However, no study has assessed the association between inflammatory cytokine levels and the severity of cataract.

The aims of this study were to evaluate the clinical, demographic characteristics, aqueous humor (AH) inflammatory cytokines during follow-up in Chinese patients diagnosed with FUS, to compare the AH inflammatory cytokines in FUS and the senile cataract eyes and to assess the association between the AH cytokine levels and the severity of posterior subcapsular cataract (PSC).

## Methods

### Study design and participants

We reviewed the medical records of 27 patients diagnosed with FUS who were admitted to our department, the Department of Ophthalmology, Beijing Chaoyang Hospital, Capital Medical University during May 2018 to May 2019. To serve as controls, 25 senile cataract patients who underwent surgery for cataract removal and intraocular lens implantation were enrolled. This study was performed in accordance with the standards of the Declaration of Helsinki. Considering the existence of invasive examination (paracentesis of anterior chamber) in this study, we have ethically recruited patients by explaining potential risk and they have voluntarily agreed for research analysis. Written informed consent was obtained from the subjects after potential risks involved with the study were explained to them. This study was approved by the Institutional Review Board of Beijing Chaoyang Hospital (No. 2018-4-3-3).

Diagnostic criteria: Diagnosis of FUS was principally based on the criteria of Kimura et al. [10]. The major criteria included the presence of (i) diffuse KPs (stellate or non-stellate), (ii) mild anterior chamber reaction defined as up to 2+ cells and flare, (iii) absence of posterior synechiae, and (iv) absence of ciliary congestion or red eye. The minor criteria for diagnosis included (i) heterochromia of the iris with/without iris depigmentary changes, (ii) presence of multiple nodules on iris, (iii) presence of vitreous opacities, and (iv) unilateral or bilateral involvement (one eye only was enrolled). Two of the four major criteria with or without the presence of minor criteria were required for diagnosis.

The demographic and clinical characteristics were obtained from each participant. Data included age, gender, ocular and medical history. 16 AH samples were obtained from patients with classical clinical signs of FUS while the disease was active. The other 11 patients did not undergo AH testing due to personal reasons. The intraocular antibody synthesis of rubella virus (RV) was confirmed by using the antibody index (AI) described in the literature [11].

### Regular ophthalmologic examinations

Regular ophthalmic examinations, including slit-lamp biomicroscopy, best-corrected visual acuity (BCVA) on the Snellen chart, intraocular pressure (IOP), and fundoscopy with dilated pupils. Some detailed ophthalmic characteristics, such as keratic precipitates, iris atrophy, iris nodules, anterior chamber reaction and vitreous reaction were also evaluated. Evaluation of aqueous cells was done by laser flare photometry (Model KOWA FM-600, Hamamatsu Factory, Japan). The main manifestation of complicated cataract in FUS patients is PSC. PSC is classified from grade 1 to 5, according to the Lens Opacities Classification System III (LOCS III) [12].

### Aqueous humor cytokine levels assessment

AH samples were collected. Patients were given topical anesthesia, and a 1-ml syringe was inserted at the peripheral cornea parallel to the iris. AH samples of 100 μL were extracted. The concentration of 6 immune mediators were measured with BD™ Cytometric Bead Array Kit (CBA), a BD FACScalibur flow cytometer and BD CBA software (Becton, Dickinson & Co, Franklin Lakes, NJ, USA): VEGF, basic fibroblast growth factor (bFGF), interleukin-6 (IL-6), interleukin-8 (IL-8), interleukin-10 (IL-10) and vascular cell adhesion molecule (VCAM). The detection thresholds of the assays were: VEGF 4.5 pg/ml, bFGF 3.4 pg/ml, IL-6 1.6 pg/ml, IL-10 0.13 pg/ml, VCAM 12.2 pg/ml, IL-8 1.2 pg/ml.

The levels of cytokines in the AH of the senile cataract group were also examined. The senile cataract group was used as the control group to analyze the elevated degree of inflammatory cytokine levels in the aqueous humor of FUS patients. Rubella virus (RV) IgG antibodies in the AH were measured quantitatively with a commercial ELISA kit (Virion/serion GmbH, Würzburg, Germany) in AH. The antibody activities were expressed in international units per mL (IU/mL). The assay was conducted according to the manufacturer’s instructions. Data analysis was performed using SERION easy base 4PL software.

### Statistical analysis

The Mann-Whitney U test were used to compare parameters between the two groups. Descriptive statistics were calculated as the median, interquartile range (IQR) for non-normally distributed variables. To assess the difference in AH cytokine levels between FUS patients and the normal controls, box plots was created to allow visualization of the data. Kendall’s tau-b rank correlation was used to evaluate associations between age, the AH cytokine levels and severity of cataract. All analyses were performed with SPSS Statistics, version 22.0 (SPSS Inc., Chicago, IL, USA), and *P* < 0.05 was considered statistically significant 2-sided.

## Results

### Demographics and symptoms

The present study included 27 patients diagnosed with FUS. 15 (55.56%) of the patients were male, 12 (44.44%) of the patients were female. The median age at diagnosis was 32.00 years old (IQR 10.00). The median follow-up time was 19.00 months (IQR 15.00). The right eye was involved in 17 patients (62.96%) and the left eye was involved in 9 patients (33.33%), while 1 patients (3.70%) had bilateral involvement. Decreased visual acuity or blurred vision were the most common complaints at presentation (16 eyes, 57.14%). 11 patients (40.74%) had no symptoms. (Table 1).

**Table 1:** Baseline demographics and symptoms of all patients

Characteristic	FUS group	Cataract group
Number of eyes	28	25
Age (years), median (IQR)	32.00 (10.00)	
Sex (male / female)	15/12	9/16
Follow-up time (years), median (IQR)	19.00 (15.00)	–
Bilateral involvement, n (%)	1 (3.70%)	–
Intraocular pressure (mmHg), median (IQR)	14.50 (3.25)	15.00 (2.00)
Ophthalmologic symptoms, n (%)		
Decreased visual acuity or blurred vision	16 (57.14%)	25 (100%)
Floaters	10 (35.71%)	0
Posterior synechiae, n (%)	1 (3.70%)	0
Systemic diseases, n (%)		
Rheumatoid arthritis, n (%)	1 (3.70%)	0
Thyroid disease, n (%)	0	0

*FUS* Fuchs uveitis syndrome, *SD* Standard deviation

**Fig. 1:**
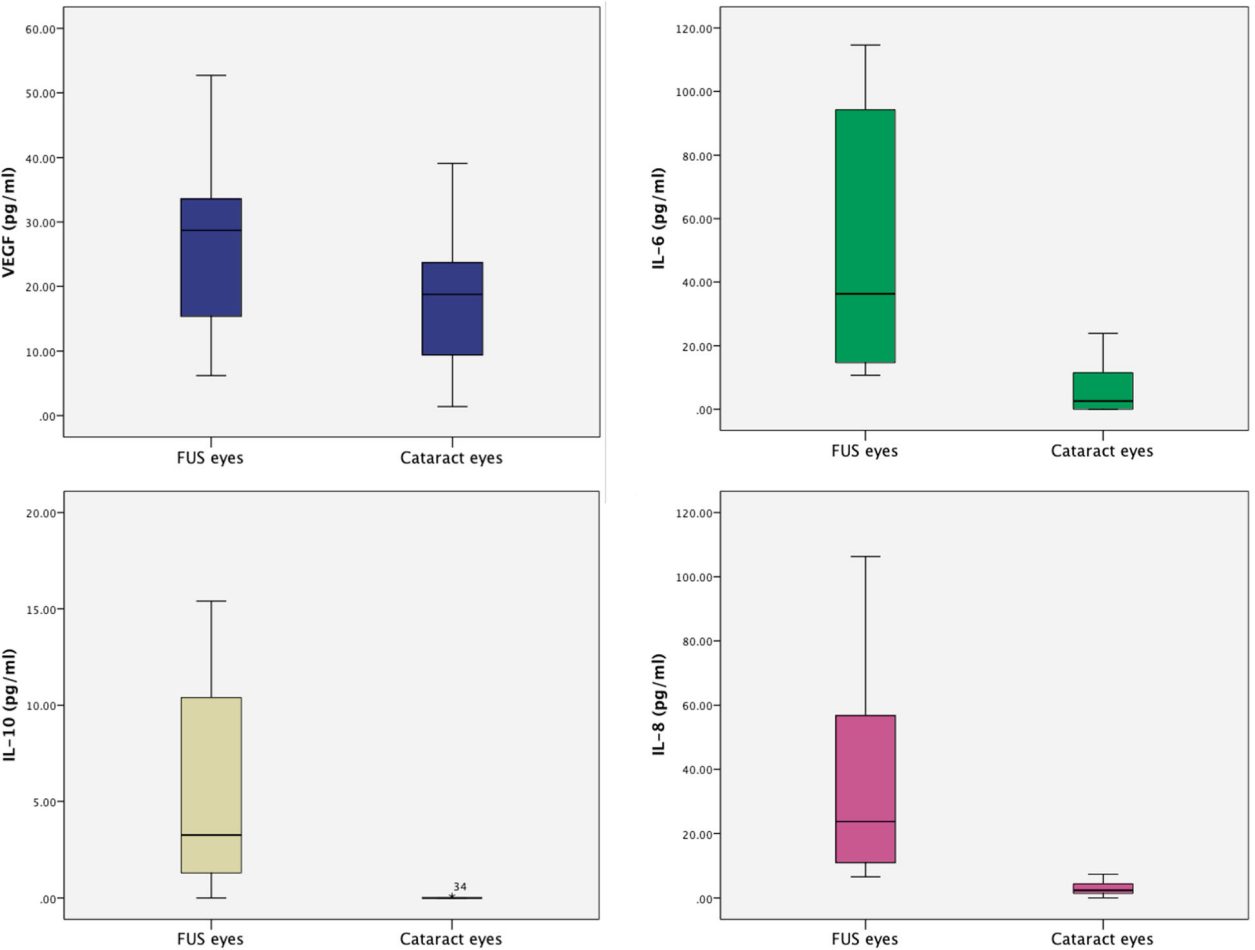
The aqueous humor cytokine levels in FUS eyes and cataract eyes

### Ocular findings

Stellate KPs were found in 16 (57.14%) FUS eyes. Anterior chamber (AC) reaction was observed in all of the affected eyes. Aqueous cells were observed in only 10 eyes, and aqueous flare was observed in 19 eyes. Although there were varying degrees of iris depigmentation in some patients, 6 of the eyes (21%) presented with heterochromia. 2 eyes (7.14%) had iris atrophy. Iris nodules were observed in 35.71% of the affected eyes. At diagnosis, 16 eyes (57.14%) presented with lens opacity: posterior subcapsular cataract (PSC), the median nuclear opalescence obtained from LOCS III was 2.00 (IQR 2.00). The duration of eyes with PSC was 18 (2.5) months, and the duration of eyes without PSC was 12 (0.5) months. Vitreous cells were noted in 7 eyes (25.00%). (Table 2).

**Table 2:** Ocular findings in 28 eyes of 27 patients at time of presentation

Finding	FUS patients
Stellate KPs, n (%)	16 (57.14%)
Anterior chamber reaction, n (%)	
Aqueous cells	10 (35.71%)
Aqueous flare	19 (67.86%)
Iris atrophy, n (%)	2 (7.14%)
Heterochromia, n (%)	6 (21.43%)
Iris nodules, n (%)	10 (35.71%)
Koeppe	8 (28.57%)
Busacca	2 (7.14%)
Lens opacity, n (%)	16 (57.14%)
LOCS III grade, median (IQR)	
PSC	2 (2)
Vitreous cells, n (%)	7 (25.00%)
Vitreous opacity, n (%)	14 (50.00%)
Mild	6 (21.43%)
Severe	8 (28.57%)

*FUS* Fuchs uveitis syndrome, *KP* Keratic precipitates, *PSC* Posterior subcapsular opacity, *LOCS III* Lens Opacities Classification System III, *SD* Standard deviation

### Complications

The most common complication was PSC (16 eyes, 57.14%), followed by epiretinal membrane (4 eyes, 14.28%) and chorioretinal lesion (3 eyes, 10.71%). Observed complications were presented in Table 3.

**Table 3:** Complications observed in patients with Fuchs’ uveitis syndrome

Complication, n (%)	FUS patients
Cataract	16 (57.14%)
Glaucoma	2 (7.14%)
Iris pigmentation on the IOL	2 (7.14%)
Glaucomatous optic disc	2 (7.14%)
Epiretinal membrane	4 (14.28%)
Chorioretinal scar	1 (3.70%)
Intravitreal hemorrhage	1 (3.70%)
Chorioretinal lesion	3 (10.71%)

*FUS* Fuchs uveitis syndrome, *IOL* Intraocular lens

### Growth factors, AH cytokines and RV antibody levels

Growth factors and AH cytokines levels of 16 FUS eyes and 25 senile cataract eyes were showed in Table 4. Statistically significant differences were found between the two groups (Fig. 1). The mean concentrations of VEGF and bFGF in FUS eyes were higher than in the cataract eyes (*P* = 0.014; *P* = 0.026). The median (IQR) of IL-6, IL-8 and IL-10 were 36.35 (88.625) pg/mL, 17.90 (47.70) pg/mL and 3.25 (10.35) pg/mL, respectively. The FUS eyes had significantly higher AH cytokine levels (IL-6, IL-8 and IL-10) compared with the cataract eyes (*P* < 0.001). Levels of VCAM in FUS eyes were higher than in cataract eyes (*P* < 0.001). Viral antibody levels of RV were also shown in Table 4. 11 (68.75%) patients had a positive outcome for intraocular antibody production against RV, with IgG more than 20 IU/mL.

**Table 4:** Cytokines and viral antibody levels in all measured aqueous humor

Mediators (pg/mL)	FUS group (*n* = 16)	Cataract group (*n* = 25)	*P* value
VEGF (pg/mL), median (IQR)	31.05 (18.625)	18.80 (17.05)	*P* = 0.014∗
bFGF (pg/mL), median (IQR)	11.30 (58.925)	0 (5.40)	*P* = 0.026∗
IL-6 (pg/mL), median (IQR)	36.35 (88.625)	2.60 (12.15)	*P* < 0.001∗
IL-8, (pg/mL), median (IQR)	17.90 (47.70)	2.40 (3.45)	*P* < 0.001∗
IL-10 (pg/mL), median (IQR)	3.25 (10.35)	0 (0)	*P* < 0.001∗
VCAM (pg/mL), median (IQR)	1706.65 (3838.275)	22.00 (77.90)	*P* < 0.001∗
Viral antibody (positive), n (%)			
RV	11 (68.75%)		

*P* values were calculated using the Mann–Whitney U test

*VEGF* Vascular endothelial growth factor, *bFGF* Basic fibroblast growth factor, *IL* Interleukin, *VCAM* Vascular cell adhesion molecule, *RV* Rubella virus, *IQR* Interquartile range

### Correlation between AH cytokine levels and severity of PSC

The main manifestation of complicated cataract in FUS patients is PSC. A correlation analysis between severity of PSC and age, levels of VEGF, bFGF, IL-6, IL-8 and IL-10 were shown in Table 5. There was a statistically significant positive correlation between the severity of PSC and IL-6, IL-8 levels (τ = 0.664 and 0.634, respectively; *P* = 0.001, *P* = 0.002, respectively). But there were no correlation between the severity of PSC and age, VEGF, bFGF and IL-10 levels (τ = 0.058, 0.117, 0.208 and 0.127, respectively; *P* > 0.05). (Table 5).

**Table 5:** Kendall’s tau-b rank correlation analyses between parameters and PSC grade

Parameters	PSC grade Kendall’s tau-b rank correlation	
	τ	*P*
Age	**0.058**	***P*** **= 0.774**
Cytokines		
VEGF	0.117	*P* = 0.565
bFGF	0.208	*P* = 0.324
IL-6	0.664	*P* = 0.001*
IL-8	0.634	*P* = 0.002*
IL-10	0.127	*P* = 0.533

*PSC* Posterior subcapsular cataract, *VEGF* Vascular endothelial growth factor, *bFGF* Basic fibroblast growth factor, *IL* Interleukin, *VCAM* Vascular cell adhesion molecule

**Fig. 2:**
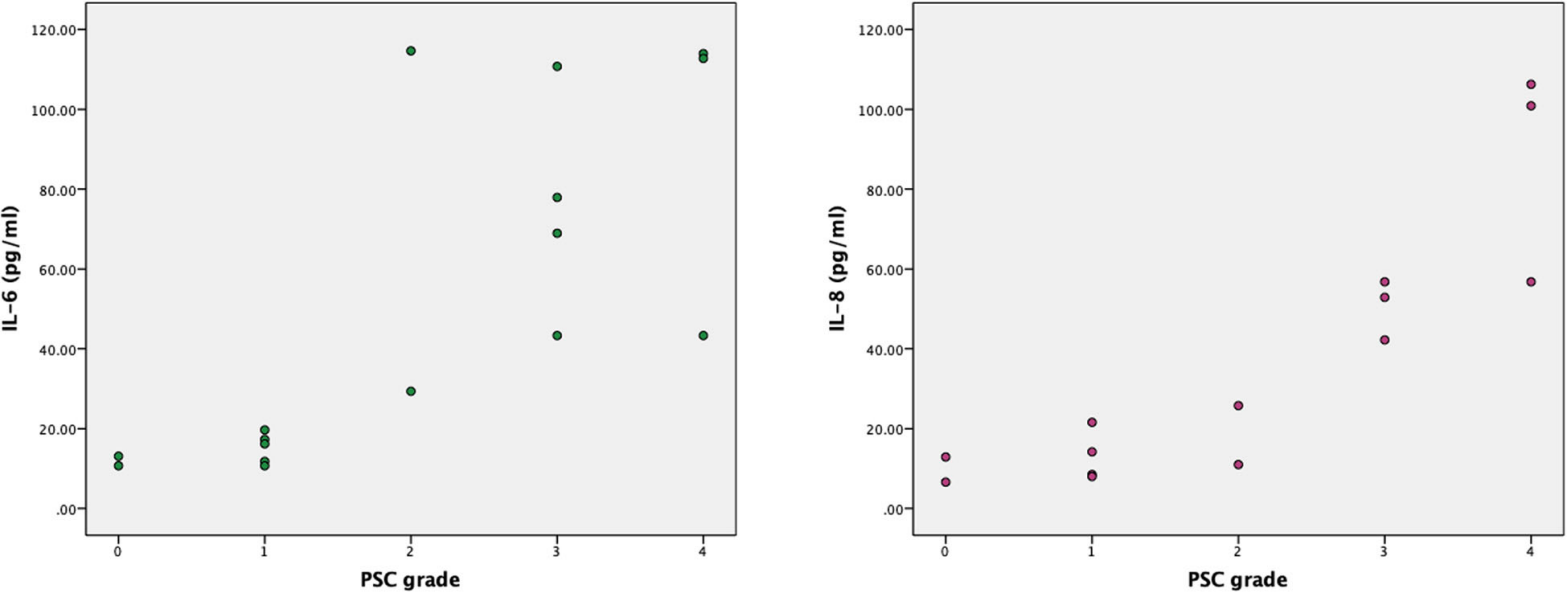
Scatter plot graph showing the relationship between AH cytokine levels and PSC grade

The severity of PSC can be classified as grade 1 to 5 according to the LOCS III grading system, as mentioned above. The number of patients with grade 5 PSC was 0. The scatter plot graph showing the relationship between IL-6 and IL-8 levels and PSC grade was shown in Fig. 2.

## Discussion

Fuchs uveitis syndrome is an unilateral chronic recurrent non-granulomatous uveitis syndrome first described in 1906 by Fuchs, and its diagnosis is determined based on clinical manifestations [1]. In this paper, the clinical manifestations and ocular signs of 27 patients were studied in detail. We found FUS patients were characterized by a mild uveitis with characteristic stellate KPs, iris heterochromia, iris nodule, complicated cataract and vitreous inflammatory reaction in this study. Immune mediators play a crucial role in specific viral intraocular inflammation. The incidence of complicated cataract in FUS patients is positively correlated with the AH inflammatory cytokine levels.

The clinical features of FUS have been described in many studies, including blurred vision [13, 14], stellate KP [13, 15, 16], iris heterochromia [14], iris nodules [17], anterior chamber and vitreous opacities [18]. FUS is the most easily misdiagnosed uveitis because a comprehensive understanding of it is lacking, there are hidden incidence and its pathological mechanisms are complex. In the present study, the most common symptoms were blurred vision and floaters. Stellate KPs were noted in 16 eyes (57%). Heterochromia was observed in 21% of affected eyes. Iris nodules were present in 36% of the affected eyes. These results are similar to the above-mentioned studies.

FUS is an unilateral chronic recurrent non-granulomatous uveitis syndrome accounts for 2 ~ 11% of all uveitis. In our study, we tried to detect the levels of cytokines in the AH of FUS patients and showed several cytokines were significantly increased compared to the controls. Vascular endothelial growth factor (VEGF) is produced by endothelial cells, activated T lymphocytes [19]. It increases vascular permeability significantly and is associated with inflammatory and immune-mediated pathology [20–22]. VEGF concentrations increased significantly in the plasma of Behçet disease (BD) patients and in the AH of patients with uveitis with associated cystoid macular edema (CME) [23, 24]. Paroli et al. [25] found VEGF levels were significantly higher in both the AH and serum of uveitis patients as compared with control subjects. Simsek et al. [26] also suggested that VEGF levels were higher in the AH of patients with FUS than healthy controls. In the present study, we found that VEGF levels were significantly higher in FUS patients compared to controls, emphasizing the possible role of VEGF in intraocular inflammatory diseases.

Ocular inflammation occurs in a variety of human disease states, inflammation is thought to be initiated by eye-reactive T cells [27, 28]. The T helper 1 (TH1) cells secrete IFN-γ, TNF-α, IL-2, IL-8 and mediate cellular immunity, activate macrophages to kill intracellular pathogens, and play an important role in immune regulation in the induction of organ-specific autoimmune diseases and anti-infective immunity. The Th2 cells secrete cytokines including IL-4, IL-5, IL-6, IL-10, IL-13 [29] and mainly support humoral immunity, resisting extracellular pathogens. Some inflammatory factors may play an important role in the pathogenesis of FUS. In our study, we detected significantly increased levels of inflammatory factors (IL-6, IL-8 and IL-10) in FUS eyes compared with senile cataract eyes.

Some researchers believed that there is a correlation between the level of intraocular inflammatory factors and IOP, and conducted studies on patients with different diseases, but came to opposite conclusions. Takai et al. found the levels of TGF-1β, IL-8, and SAA were positively correlated with IOP in patients with open-angle glaucoma, indicating that cytokine networks in aqueous humor may have critical roles in IOP elevations [30]. Pohlmann et al. [31] examined clinical characteristics of Posner-Schlossman-Syndrome (PSS) and Fuchs’ Uveitis (FU) patients and found that immune mediators correlate negatively with IOP in the PSS patients. They also substantiated a similar composition of cytokines in PSS patients suffering from ocular hypertension and thus offers a potential explanation model for a negative correlation between mediators and IOP. We also analyzed the IOP level and did not detect significant difference between the two groups. Perhaps there is a correlation between the IOP level and the level of inflammatory factors in FUS patients. But we did not did not get similar results may be limited by small sample size, future study with large sample size should be carried out to confirm the correlation between IOP and the intraocular cytokines.

IL-6 can stimulate the proliferation, differentiation and function of cells involved in immune responses and play an important role in anti-infective immune response. Pohlmann D et al. [31] found that IL-6 was significantly higher in FUS and PSS patients compared to controls. In another study, T. gondii patients had twofold higher levels of IL-6 as compared to healthy subjects, which seems to confirm the presence of an inflammatory state [32]. Our study also found an increase in the level of IL-6 in FUS patients.

High IL-10 levels are mainly associated with active infectious uveitis and are considered to be important in early stages of infection [33]. In the previous study, a significant positive correlation between VKH disease and high levels of IL-10 was found by El-Asrar et al. [34]. Sijssens et al. [33] found that high IL-10 levels are associated with active infectious uveitis are considered to be important in early stage of infection. Joanna et al. also found the level of IL-10 to be fivefold higher in the course of toxoplasmosis than in healthy controls [32]. In FUS patients, increased levels of IL-10 are assumed to imply a distinctively acute inflammation triggered by RV. In our study, we detected significantly increased levels of IL-10 in FUS patients compared with normal eyes.

IL-8 has a role in chemokine functions. Mo JS et al. [35] found that IL-8 played a key role in the pathogenesis of intraocular inflammation in a rabbit model of uveitis. In the studies of humans, high levels of IL-8 have been detected in the aqueous humor of patients with acute anterior uveitis [36], Behçet disease (BD) or Vogt-Koyanagi-Harada (VKH) disease [37, 38]. Simsek et al. [26] also emphasized IL-8 levels were significantly higher in the aqueous humor of patients with FUS than in the AH of control subjects. We also found high IL-8 levels in FUS patients. IL-8 is a cytokine secreted by TH1 cells, which mainly mediate the production of immune antibodies related to the organism’s local inflammatory reaction and participate in cellular immunity and delayed hypersensitivity inflammation. IL-8 may act as a marker for inflammation in the AH in FUS. In our study, we detected immune mediators (IL-6, IL-8 and IL-10) increased significantly in FUS patients compared with cataract eyes, suggesting that IL-6, IL-10 and IL-8 may play an important role in anti-infective immune response and contribute to the viral response in FUS.

Cataract is a common complication of chronic or recurrent uveitis and is a sequelae to chronic intraocular inflammation and topical corticosteroid therapy. Cataracts occur in many types of uveitis, such as juvenile idiopathic arthritis-associated uveitis, BD, VKH and ocular toxoplasmosis. Cataract occurs in 17–36% of ocular BD patients and the most frequent complication is cataract [39, 40]. In VKH, cataract is the most common complication, with a prevalence of about 40%. The expression of pro-inflammatory cytokines in the cataract formation of non-infectious uveitis is gradually understood. Recurrent uveitis attacks may lead to lens permeability, then result in cataract [41]. Previous studies have emphasized that cataract is the most common complications in FUS patients [13, 16, 42]. Tugal-Tutkun et al. [16] found a 56% risk of cataract formation in FUS patients over their 8-year follow-up period. Yang et al. [13] found cataract was appeared in 70.7% of their FUS patients. Similarly, cataract was observed in 57.14% of patients in our study. The use of hormonal drugs to treat of inflammation also promotes the development of cataract.

Our research speculated that the occurrence of complicated cataract in FUS patients may be closely related to intraocular inflammation. Previous studies have confirmed that there is a correlation between inflammatory factors in the AH of uveitis patients and the occurrence of cataract. Tumor necrosis factor-α (TNF-α) can increase the mRNA level of laminin and type IV collagen, cause lens capsule fibrosis and the proliferation and migration of lens epithelial cells (LEC), thereby promoting the development of cataract. Transforming growth factor-β (TGF-β), which can induce lens cell pathology and lead to fibrotic cataract formation [43]. Recently, few studies have explored the association between cataract in FUS patients and the high expression of inflammatory cytokines in the AH. Thus, the relationship between the levels of intraocular cytokines and cataract in FUS is evaluated in this study.

Previous studies have confirmed that IL-1 can promote the proliferation of LEC and collagen synthesis, then resulting in the formation of cataract [44]. There are also some researches revealed that IL-1β can promote the expression of IL-6 and IL-8 through different pathways. Hu et al. [45] first reported the expression and secretion of IL-6 by human uveal melanocytes (UMs) and found IL-1β increases expression and secretion of IL-6 via the p38 MAPK/NF-kappaB pathway. Liu et al. [46] revealed that IL-1β induces IL-6 production in Müller cells by activation of IL-6 promoter activity predominantly through the p38 MAPK/NF-κB pathway. IL-1β potently stimulates IL-8 expression in Müller cells mainly through the p38 MAPK and ERK1/2 pathways [47]. Chang et al. [48] also found the important role of IL-1β in pulpal inflammatory responses via stimulation of IL-8 and ICAM-1 expression and secretion. The triggering effects of the inflammatory mediators, such as IL-1 and IL-6, may have an early stimulating effect on posterior capsular opacification (PCO) formation, and the actions of IL-1 may be mediated through the production of IL-6 [44].

The correlations between the severity of cataract and age, and AH cytokines (VEGF, bFGF, IL-6, IL-8 and IL-10) were explored in the present study. There was a statistically significant positive correlation between the severity of cataract in FUS patients and IL-6 and IL-8 level. These results confirmed that the occurrence of complicated cataract in FUS patients is positively correlated with the high expression of inflammatory factors. Further studies are needed to confirm their exact effect on the course of uveitis and complicated cataract.

The study has also several limitations. Firstly, this study only analyzed some inflammatory factors (IL-6, IL-10 and IL-8) in the AH of FUS patients, but did not explore the progression of other inflammatory factors in the AH, such as IL-1, TNF-α and TGF-β during the course of FUS, and their associations with cataract formation. Secondly, the sample size of this study was relatively small, which may have limited the statistical strength of the analysis.

## Conclusions

In conclusion, the present results showed that in the current study the expression of inflammatory factors in the AH of FUS patients is significantly increased, and further revealed a positive correlation between the levels of IL-6 and IL-8 levels in the AH and the PSC grade in FUS, which may help to explain the early formation of cataract in FUS.

## Supplementary Information

**Additional file 1.** Demographic and clinical data in this study.

## Data Availability

A supplemental material which included the primary data has been uploaded accordingly (see Additional file 1).

## Abbreviations

AH: Aqueous humor
AI: Antibody index
BCVA: Best-corrected visual acuity
BD: Behçet disease
EGF: Epidermal growth factor
FUS: Fuchs uveitis syndrome
IL: Interleukin
IOP: Intraocular pressure
KP: Keratic precipitates
LEC: Lens epithelial cells
MMP: Matrix metalloproteinase
PSC: Posterior subcapsular cataract
PSS: Posner–Schlossman Syndrome
RV: Rubella virus
TGF: Transforming growth factor
TNF: Tumor necrosis factor
TH: T helper
Ums: Human uveal melanocytes
VCAM: Vascular cell adhesion molecule
VEGF: Vascular endothelial growth factor
VKH: Vogt–Koyanagi–Harada syndrome
